# Complete Sequence of the Intronless Mitochondrial Genome of the Saccharomyces cerevisiae Strain CW252

**DOI:** 10.1128/genomeA.00219-18

**Published:** 2018-04-26

**Authors:** Delphine Naquin, Cristina Panozzo, Geneviève Dujardin, Erwin van Dijk, Yves d’Aubenton-Carafa, Claude Thermes

**Affiliations:** a I2BC Next-Generation Sequencing Facility, Institut de Biologie Intégrative de la Cellule, UMR9198, CNRS CEA Université Paris-Sud, Université Paris-Saclay, Gif-sur-Yvette, France; b Biogenesis and Functioning of Mitochondrial Oxphos Complexes, Institut de Biologie Intégrative de la Cellule, UMR9198, CNRS CEA Université Paris-Sud, Université Paris-Saclay, Gif-sur-Yvette, France

## Abstract

The mitochondrial genomes of Saccharomyces cerevisiae strains contain up to 13 introns. An intronless recombinant genome introduced into the nuclear background of S. cerevisiae strain W303 gave the S. cerevisiae CW252 strain, which is used to model mitochondrial respiratory pathologies. The complete sequence of this mitochondrial genome was obtained using a hybrid assembling methodology.

## GENOME ANNOUNCEMENT

The mitochondrial genome of the Saccharomyces cerevisiae CW252 strain (1, 2) was sequenced using both Illumina short-read and MinION long-read sequencing technologies. In the first step, reads issued from 2 runs of the Illumina MiSeq platform and GAIIx system with a quality score greater than 30 were purified by mapping with TopHat2 (3) on the mitochondrial genome of the S. cerevisiae S288c parent strain; they were then sampled by Trinity (4) to obtain an estimated coverage of 30×. An assembly of the genome obtained by Velvet (5) with a k-mer size of 55 produced 34 contigs (*N*_50_, 3.7 kb), which were subsequently scaffolded by SSPACE (6) to obtain 10 contigs (*N*_50_, 57.5 kb). Some of these scaffolds seemed to be chimeric, since a mapping of the original paired-end reads on this assembly by BWA (7) displayed only 85% success. A correction step was then performed by REAPR (8) to break the chimeric scaffolds, producing a coherent assembly of 38 scaffolds of good quality (*N*_50_, 2 kb). Examination of the mapping of these scaffolds on the S288c mitochondrial genome showed that many of the gaps between scaffolds contained GC-rich clusters which could hamper Illumina sequencing (9). In a second step, two-dimensional (2D) base calling of a single run on a MinION R9.4 flow cell, using Metrichor, produced 29,276 reads with a mean length of 4 kb and a total length of 120,259 kb. The correction, trimming, and assembly of these reads by Canu version 1.4 (10) produced 2 contigs, one of 84 kb corresponding to the mitochondrial genome, and another of 34 kb corresponding to contamination from the nuclear genome. The mitochondrial contig was first trimmed at both ends to remove overlapping extremities. To improve the quality of the MinION sequencing, the assembly was polished using two strategies, (i) the Pilon version 1.21 hybrid method (11) to directly correct the assembly using the Illumina reads by five iterative correction steps and (ii) a prior polishing of the sequence by Nanopolish (12), followed by four correction steps by Pilon. The second method gave the best results, with approximately 2-fold fewer nucleotide variations than the Illumina scaffolds, and the assembly was retained as a bona fide backbone. To improve the final assembly, the scaffolds issued from the Illumina assembly were replaced on this backbone, and the resulting mitochondrial genome was then annotated. The unique contig sequence was 70,523 bp long, and no nucleotide variation was shown in several regions spanning 8,500 nucleotides (nt) sequenced by the Sanger method. This genome displays the mosaic structure of its construction. Particularly, the 3ʹ sequence of the *COX3* gene is different from that of its S288C parent strain and corresponds to the sequence of the S. cerevisiae D273-10B strain (13).

### Accession number(s).

The genome sequence is available in GenBank with accession number MG916964.
